# Vitamin D and calcium, together and separately, play roles in female reproductive performance

**DOI:** 10.1038/s41598-022-14708-7

**Published:** 2022-06-21

**Authors:** Hengameh Safari, Mehdi Hajian, Mohammad Hossein Nasr-Esfahani, Mohsen Forouzanfar, Joël R. Drevet

**Affiliations:** 1 Department of Biology, Shiraz Branch, Islamic Azad University, Shiraz, Iran; 2 Department of Animal Biotechnology, Reproductive Biomedicine Research Center, Royan Institute for Biotechnology, ACECR, Isfahan, Iran; 3 GReD Institute, Faculté de Médecine, Université Clermont Auvergne-INSERM-CNRS, Clermont-Ferrand, France

**Keywords:** Physiology, Embryology

## Abstract

Vitamin D (VD) deficiency reduces the chances of successful fertilization; however, it remains to be validated whether this effect is dependent or not on calcium. To address this question, we generated several situation using a mouse model in which VD content was either increased or decreased in a normo or hypocalcemia context. After the measurement of serum 25-hydroxyvitamin D_2_, calcium and phosphorus levels, an analysis was carried out in terms of oocytes maturation as well as reproductive performance. VD overdose, despite the fact that it resulted in an increased number of mature oocytes, reduced developmental competence and offspring survival. VD deficiency (VDD), on the contrary, reduced the number and percentage of mature oocytes, blastocyst rate, as well as fertility rate and offspring survival. Hypo-calcemia when VD levels were normal, had a similar effect than VDD. The effects of VDD were reversed by a diet that corrected calcium level. Therefore, both VD overdose (in a context of normal calcium level) VD deficiency as well as hypo-calcemia have an effect on female reproductive function. In conclusion, although closely related, VD and calcium act in part independently of each other in defining the “optimum” for female reproductive performance.

## Introduction

Vitamin D (VD) is an essential micronutrient that not only plays a major role in calcium homeostasis and the health of the body's skeletal system, but also affects the function of many organs in mammals. VD deficiency (defined as a serum 25-hydroxyvitamin D level below 20 ng/ml) is a common condition estimated to affect approximately 50% of the world's population and is a major worldwide public health problem^1,2^. VD deficiency is associated with many diseases, including diseases of the immune and cardiovascular systems, as well as diabetes, obesity, cancer, and infertility in both male and female^2,3^. Specifically, in recent years, female infertility has been partially suspected to be associated with VD deficiency^4^.

VD acts through two different and distinct mechanisms: a calciotropic effect and a non-calciotropic effect^5^. The calciotropic function of VD contributes to the maintenance of normal serum calcium and phosphorus concentrations by stimulating the small intestine to absorb these minerals from food^6^. The non-calciotropic effect of VD is less clear^7^, and is primarily induced by the nuclear VD receptor (VDR) which acts in concert with the retinoid-X-receptor (RXR), forming a heterodimer^8^. The VDR-RXR heterodimer binds to VD-responsive elements (VDREs) located in the promoter region of some target genes, whose transcription it regulates^9,10^. VDR is expressed in female reproductive tissues, including the ovaries, endometrium, fallopian tubes, and placenta in both humans and mice^11–13^.

Although VD is implicated in many pathological situations, it is not yet clear whether its effects primarily stem from its calciotropic or non-calciotropic actions. In an attempt to clarify this issue, in a previous report, we took advantage of an animal model in which VD deficiency was induced while calcium and phosphorus levels were maintained. In this context, we showed that, independently of its calciotropic effect, VD deficiency affected the reproductive performance of males, leading to the production of defective spermatozoa with reduced chromatin integrity^14^. Here, we present the data obtained from the female side. With the same rationale, we took advantage of animal models in which we were able to generate the following situations: 1) control, 2) sham, 3) VD overdose and normo-calcemia (VD^++^Ca^+^), 4) VD deficient and hypo-calcemia (VD^−^Ca^−^), 5) VD deficient and normo-calcemia (VD^−^Ca^+^) and 6) VD normal and hypo-calcemia (VD^+^Ca^−^).

In these different situations, we then evaluated the reproductive performance of female mice by examining various parameters of oocyte production and fetal development after mating. Our objective was to attribute more specific effects to VD and calcium. These effects, if translatable to the clinic, could potentially contribute to the development of new therapeutic approaches.

## Results

**Figure 1:**
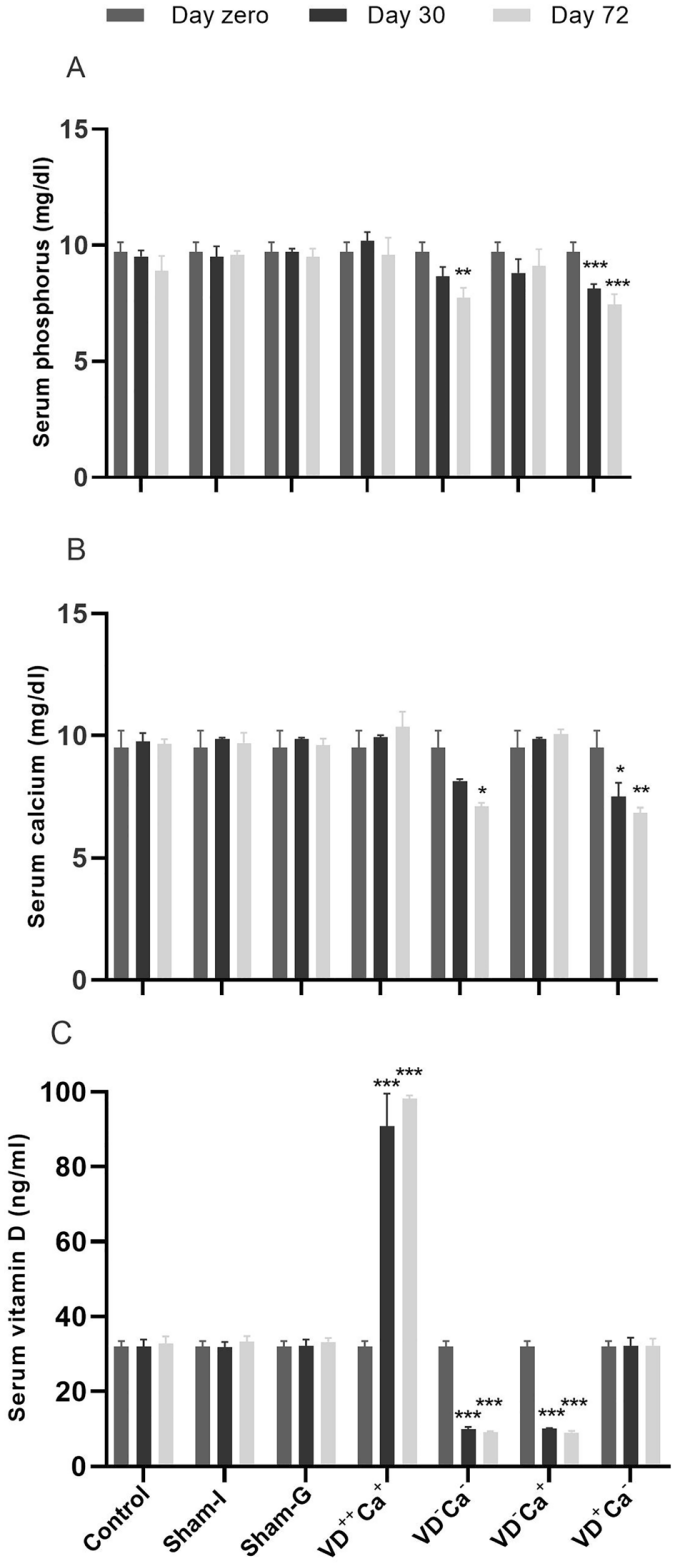
Serum 25-hydroxyvitamin D_2 _(**C**) calcium (**B**), and phosphorus (**A**) content at days 0, 30, and 72 in the different groups of mice. *p < 0.05, **p < 0.001, and ***p < 0.0001.

VD overdosing (VD^++^Ca^+^) was evidenced by the fact that the serum 25-hydroxyvitamin D_2_ level, which was approximately 32.8 ng/ml in control animals, increased to 90.8 and 98.7 ng/ml on day 30 and 72, respectively (Fig. 1C). In a logical way, despite VD overdosing was performed as part of a calcium-deficient diet, it was not accompanied by a significant reduction in serum calcium level because of its calciotrophic effect (Fig. 1B). Similarly, paricalcitol-induced VD deficiency in animals on a normal calcium diet (VD^−^Ca^−^) was evidenced by the fact that serum VD levels were decreased to 9.9 and 9.2 ng/ml on days 30 and 72, respectively, compared with control and sham animals. As previously reported^14^ paricalcitol-mediated VD deficiency was accompanied by a reduction in phosphorus and calcium levels that was not significant until day 72 (Fig. 1A,B). Paricalcitol-mediated VD deficiency was also confirmed in animals fed a calcium-enriched diet (VD^−^Ca^+^) as the serum VD level at day 30 and 72 was 10 ng/ml and 9 ng/ml, respectively. Serum calcium level returned to normal (control) values in animals fed a Ca-enriched diet (VD^−^Ca^+^). Finally, animals fed a Ca-deficient diet but with normal serum VD level (VD^+^Ca^−^) had significantly decreased serum calcium level on days 30 and 72 (Fig. 1A,B). Interestingly, the decrease in serum calcium level generated by either VD deficiency or a Ca-deficient diet was of the same magnitude, making the models comparable.

### Oocyte number and maturity in the different animal groups`

**Figure 2:**
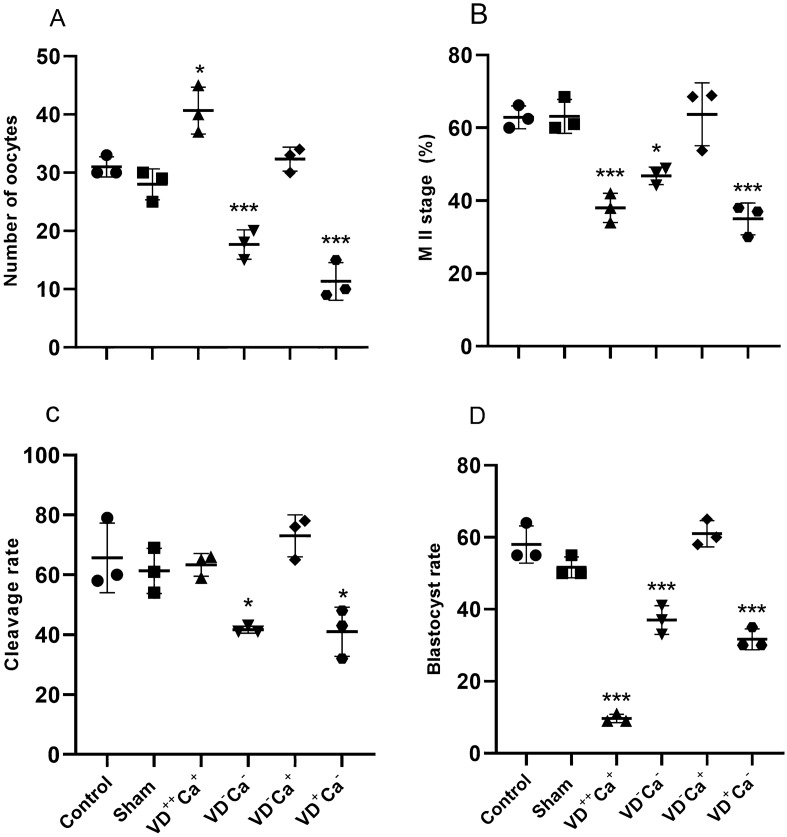
Effect of treatments on: (**A**) number of oocytes collected, (**B**) percentage of MII mature oocytes, (**C**) cleavage rate and (**D**) blastocysts rate. *p < 0.05, **p < 0.001, and ***p < 0.0001.

Mice subjected to a VD overdose (VD^++^Ca^+^) showed significantly higher mean oocyte numbers compared with control and sham groups (Fig. 2A). In comparison, animals with hypo-calcemia, irrespective of VD deficiency [(VD^−^Ca^−^) or (VD^+^Ca^−^)], had significantly reduced mean oocyte numbers. It is therefore assumed that the recorded variation in mean oocyte number is primarily related to VD content. However, in the group of VD deficiency animals reared on a calcium-enriched diet (VD^−^Ca^+^), the mean oocyte number returned to the level of the control and sham groups (Fig. 2A), suggesting that serum calcium concentration also controls the oocyte number. This is confirmed by the observation that in the last group of animals subjected to hypo-calcemia in a context of normal VD content (VD^+^Ca^−^), the mean number of oocytes was significantly reduced. Figure 2B shows that the mean number of oocytes reaching MII stage was significantly lower for all treatments, except for the VD deficient group receiving calcium supplementation (VD^−^Ca^+^) (Fig. 2B). This suggests that VDD alone (when calcium level are normal) does not influence oocyte maturation and that calcium is the more potent factor controlling oocyte maturation.

### Cleavage and blastocyst rates in the different animal groups

VD overdose (VD^++^Ca^+^) appeared to have no effect on oocyte cleavage rate, whereas it had a strong reducing effect on blastocyst rate (Fig. 2C,D). VD deficiency had a small but significant reducing effect on oocyte cleavage and blastocyst rates, which was reversed when calcium supplementation was performed (Fig. 2D,C). Hypo-calcemia in normal VD (VD^+^Ca^−^) had a significant effect on both oocyte and blastocyst cleavage rates.

### Mating and fertility rates in the different animal groups

Comparison of mating and fertility ratios between groups of animals revealed that an overdose of VD (VD^++^Ca^+^) had no effect on mating ratio but resulted in a significant reduction of the fertility ratio (Table 1). VD deficiency in a context of a diet containing a standard level of calcium (VD^−^Ca^−^) decreased mating and fertility ratios (Table 1). Interestingly, when VD deficiency was performed in a setting where the serum calcium level was corrected (VD^−^Ca^+^), both ratios returned to the levels of the control/sham groups. Consistent with the fact that serum hypo-calcemia had a significant effect on both ratios assessed, we recorded the strongest negative impact on fertility and mating ratios in the hypo-calcemic group of animals, in which the serum VD level was normal (VD^+^Ca^−^) (Table 1).

**Table 1:** Effect of vitamin D deficiency/overdose and calcium deficiency on mating and fertility rates.

Group	Control	Sham-I	VD^++^Ca^+^	VD^−^Ca^−^	VD^−^Ca^+^	VD^+^Ca^−^
Total number of mice	12	12	12	12	12	12
Mean number of days*	4 ± 0.44	4 ± 0.44	4 ± 0.22	5.2 ± 0.44	4 ± 0.22	6.5 ± 0.5
Number of pregnant mice	12	12	12	10	12	10
Mating ratio	0.25	0.25	0.25	0.16^a^	0.25	0.13^a^
No of pregnant mice giving birth 12	12	7	10	12	6	
Fertility ratio	0.25	0.25	0.14^b^	0.16^c^	0.25	0.08^a^

The groups were (1) control, (2) sham, (3) VD overdose and normo-calcemia (VD^++^Ca^+^), (4) VD deficient and hypo-calcemia (VD^−^Ca^−^), (5) VD deficient and normo-calcemia (VD^−^Ca^+^) and (6) VD normal and hypo-calcemia (VD^+^Ca^−^).In each group, 2 female mice were mated with 1 male mouse and the average number of days required for each mouse to become plug positive was recorded. The mating ratio is equivalent to number of animals multiplied by the average number of days required to become pregnant. The fertility ratio is equivalent to the number of pregnant females that give birth divided by the total number of mating days. Dunnett's multiple comparisons test was used to analyze the significance of the fertility and mating rates .^c^*p* < 0.05, ^b^*p* < 0.001, and ^a^*p* < 0.0001. *Mean number of days ± standard deviation.

### Time lag between mating/parturition, pup number and survival in the different animal groups

**Figure 3:**
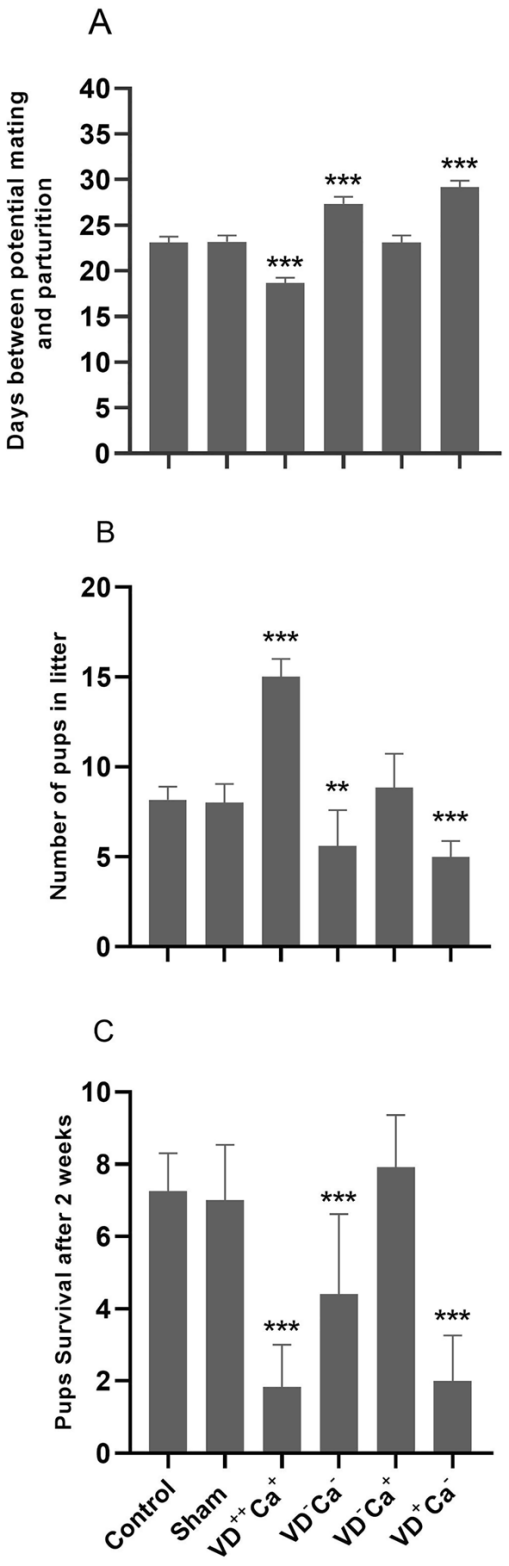
Effect of treatments on: (**A**) time from mating to parturition, (**B**) litter size and (**C**) survival rate. *p < 0.05, **p < 0.001, and ***p < 0.0001.

Assuming that the estrous cycle and gestation last 4–5 days and 18–21 days, respectively, a normal time from mating to parturition of about 22–26 days can be expected. This is consistent with the values obtained in the control and sham animal groups (23 and 23.1 days, respectively). In the group of mice VD overdosed (VD^++^Ca^+^), we recorded a significant reduction in the time from mating to parturition, which fell to 18.6 days (Fig. 3A). In contrast, in the group of VD deficient animals with hypo-calcemia (VD^−^Ca^−^) this duration was increased to 27.3, an increase that was abrogated (as it returned to 23 days) when serum calcium level was restored (VD^−^Ca^+^) (Fig. 3A). Confirming the importance of calcium in controlling the time from mating to parturition, the hypo-calcemic group in which VD was normal (VD^+^Ca^−^) showed a significant increase to 28.6 days (Fig. 3A). Looking at the average litter size, the control and sham groups had 8.1 and 8.8 pups *per* litter, respectively (Fig. 3B). A strong increase in mean litter size was recorded in the VD overdosed group (VD^++^Ca^+^) which reached 15 pups *per* litter (Fig. 3B). In contrast, in the group of VD-deficient animals fed a standard level of calcium (VD^−^Ca^−^), a significant reduction in mean litter size was observed to 5.6 pups *per* litter (Fig. 3B). This VD deficiency effect on litter size was reversed when the VD deficient animal group was supplemented with calcium (VD^−^Ca^+^) (Fig. 3B). Confirming the importance of serum calcium level in determining litter size, the group of calcium-deficient animals with normal serum VD (VD^+^Ca^−^) also showed a significant reduction in mean litter size to 5 pups/litter (Fig. 3B). Considering pup survival 2 weeks after parturition, the mean number of surviving pups in the control and sham groups was 7.2 and 7, respectively (Fig. 3C). In VD overdosed mice (VD^++^Ca^+^), a sharp reduction in the mean number of surviving pups was recorded, as it fell to 1.8 pups/litter (Fig. 3C). In VD deficient mice fed standard level of calcium (VD^−^Ca^−^), we also recorded a reduction in the average number of surviving pups, but to a lesser extent (5 pups/litter; Fig. 3C). When calcium level was restored (VD^−^Ca^+^) the average number of surviving pups *per* litter returned to that recorded for the control and sham groups (7.7; see Fig. 3C). In normal serum VD content with hypo-calcemia animals (VD^+^Ca^−^), a strong and significant reduction in the mean number of surviving pups (2 pups *per* litter) was also observed (Fig. 3C).

## Discussion

Before the evaluation of each group of animals, we validated the animal models and treatments performed in terms of serum calcium, phosphorus, and 25-hydroxyvitamin D_2_ content at days 0, 30, and 72. This validation confirmed that animals in which VD deficiency was induced by paricalcitol injections showed a significant reduction in serum calcium and phosphorus levels when fed a standard diet (VD^−^Ca^−^), a situation that was reversed when VD deficiency animals were supplemented with calcium (VD^−^Ca^+^). VD overdosing in the context of low calcium intake resulted in normal serum calcium/phosphorus levels due to the classical calciotrophic effect mediated by VD^15^. To complete the models used, we generated a situation in which serum VD content was normal but in a context of hypo-calcemia. These different situations allowed us to dissociate serum VD content (overdose, normal and deficient) from hypo-calcemia condition.

Our data reveal that a VD overdose has a strong positive effect on the number of oocytes retrieved after ovarian stimulation, at the expense of their efficient maturation, as evidenced by the significant reduction of oocytes reaching MII and blastocyst stages. Since serum calcium/phosphorus levels are normal in this group of animals, we assume that this is a VD-mediated effect. This is somehow in agreement with the work of Lee et al. who showed that VD supplementation increases mitochondrial DNA copy number, biogenesis, and membrane integrity^16^, in addition to regulating the expression of genes related to antioxidant and anti-apoptotic capacity in a PCOS mouse granulosa cell model resulting in an increase in oocyte number^17^.

On the contrary, in case of VD deficiency associated with hypo-calcemia, we obtained a significant reduction in oocytes retrieved after ovarian stimulation, as well as in their ability to mature. This was reversed when the serum calcium level was normalized, demonstrating that the serum calcium level also plays a critical role in determining the oocyte number and maturation. This was confirmed by the observation that diet-induced hypo-calcemia in the context of normal serum 25-hydroxyvitamin D_2_ caused a significant negative effect on the number of recovered oocytes as well as a significant decrease in their ability to mature. Therefore, VD overdose and calcium have partly independent quantitative and qualitative effects on oocyte production. Here we confirm the work of Sun et al.^18^ who reported that VD-deficient mice had follicular developmental defects that were attributed to low serum calcium/phosphorus levels because these were completely reversed by calcium/phosphorus supplementation. In addition, they also reported that VD deficiency caused changes in serum FSH, LH and estradiol levels that were normalized by calcium/phosphorus supplementation^19^; hormones that were not assessed in this study due to limited amounts of serum for all bio-chemical assessments. Working on a different mouse model of VD deficiency (via VD receptor knockout), Johnson et al.^20^ reported that calcium/phosphorus supplementation only partially restored estradiol levels, a difference that may be due to the specificity of each animal model. If transposable to humans, these observations could have important implications for ART. In case of low serum 25-hydroxyvitamin D_2_, which is quite common as mentioned above, calcium/phosphorus or VD supplementation could have beneficial effects on oocyte recovery and maturation capacity. This is consistent with the conclusion of a meta-analysis^21^ reporting that VD supplementation significantly increases AMH levels, a marker of ovarian reserve. However, this conclusion is not totally consensual as more recently Estes et al.^22^ did not show a beneficial effect of VD supplementation on AMH level and oocyte number. As suggested very recently by Bednarska-Czerwinska et al.^23^, these conflicting data could be explained by a dual relationship existing between serum AMH and 25-hydroxyvitamin D_2_ that would be dependent on serum 25-hydroxyvitamin D_2_ concentration. When the VD concentration in serum is below 30 ng/ml, it has been proposed that a negative relationship exists between AMH and VD, whereas above this concentration, this effect is abrogated and even reversed^23–25^. This could partly explain the increased number of oocytes obtained in our group of VD-overdosed animals. It could also explain the ambiguity in the existing literature regarding the effect of AMH and VD on oocyte number. Such behavior may have a molecular rationale, since during follicular activation, AMH suppresses follicular activation and maturation, a role attributed to the highly specific AMHR-II receptors^26,27^. As VD has been shown to decrease AMHR-II expression^24,28^, it should therefore promote oocyte activation and maturation^29,30^. This is consistent with our observations in the VD-overdosed and VD-deficient mice groups (Fig. 2). However, it is also known that the AMH promoter contains a functional VD-sensitive element (VDRE) that stimulates AMH expression^31^. Therefore, it is expected that, on the one hand, VD promotes oocyte activation and maturation through its down-regulation of AMHR-II receptors and, on the other hand, VD inhibits oocyte activation and maturation through its suppressive action on AMH^32^. This is also in agreement with our observation of reduced oocyte activation/maturation in VD-deficient animals with hypo-calcemia (VD^−^Ca^−^) (Fig. 2). Thus, depending on the VD sensitivity threshold of these antagonistic actions, it is not surprising to observe a dual effect on the follicular response.

The comparison of the different animal models generated here is interesting because it shows that VD alone, and calcium alone each modulates the number, activation and maturation of oocytes. It also shows that in the VD-deficient situation, when the calcium level is corrected, the number and activation/maturation of oocytes return to normal, highlighting the prominent role of serum calcium in follicular activity. This study also shows that VD supplementation (in the context of normal serum calcium level) can increase ovarian reserve and litter size, but does not improve oocyte maturation and live birth rate, calling into question the therapeutic value of VD supplementation outside of a clear situation of VD deficiency. However, it is important to keep in mind that caring for several pups depends also on the intrinsic capacity of each animal and that this question would have had to be answered through foster care, which was outside the scope of this study. When VD deficiency is present, our data clearly show that restoration of serum calcium level normalizes all measured parameters from oocyte number, activation, maturation to their ability to develop into viable offspring. Thus, calcium seems to be the most critical factor with respect to the parameters evaluated, since hypocalcemia in a context of normal serum VD negatively affects each of the parameters measured.

### Conclusions

Our data suggest that VD has a stimulatory effect on folliculogenesis but a negative effect on oocyte maturation and reproductive performance. Whether these antagonistic effects are due to genomic or non-genomic actions remains to be determined. In accordance with our results, Halloran and DeLuca also demonstrated earlier that VD deficiency in female rats reduced mating efficiency, litter size, and overall fertility^33^. Our data are also consistent with the delayed neonatal growth observed between days 6 and 15 postpartum when pups are fed by a VD-deficient mother^20,34^. Our work shows that serum calcium level exerts its control throughout the ovarian gametogenetic process as well as in the developing embryo. Overall, this work underscores the importance of optimal serum VD and calcium levels in female reproduction.

## Materials and methods

### Ethics and animals

All procedures were approved by the Institutional Review Board of Royan Institute, Tehran, Iran (IR.ACECR.ROYAN.REC.1399.083). All animal experiments were conducted in compliance with the ethical guidelines established by the Institutional Ethics Committee of Royan Institute. Also, the manuscript follows the recommendations in the ARRIVE guidelines.

### Media and reagents

All chemicals and media were obtained from Sigma Aldrich Chemical Co. (St. Louis, MO) and Gibco (Grand Island, NY), respectively, unless stated. The Institutional Review Board and Institutional Ethical Committee of the Royan Institute approved all animal care protocols and the proposal (99000009). In addition, all methods used in this study were handled according to the guidelines and regulations provided by the Institutional Review Board and Institutional Ethical Committee of the Royan Institute.

### Experimental design

**Figure 4:**
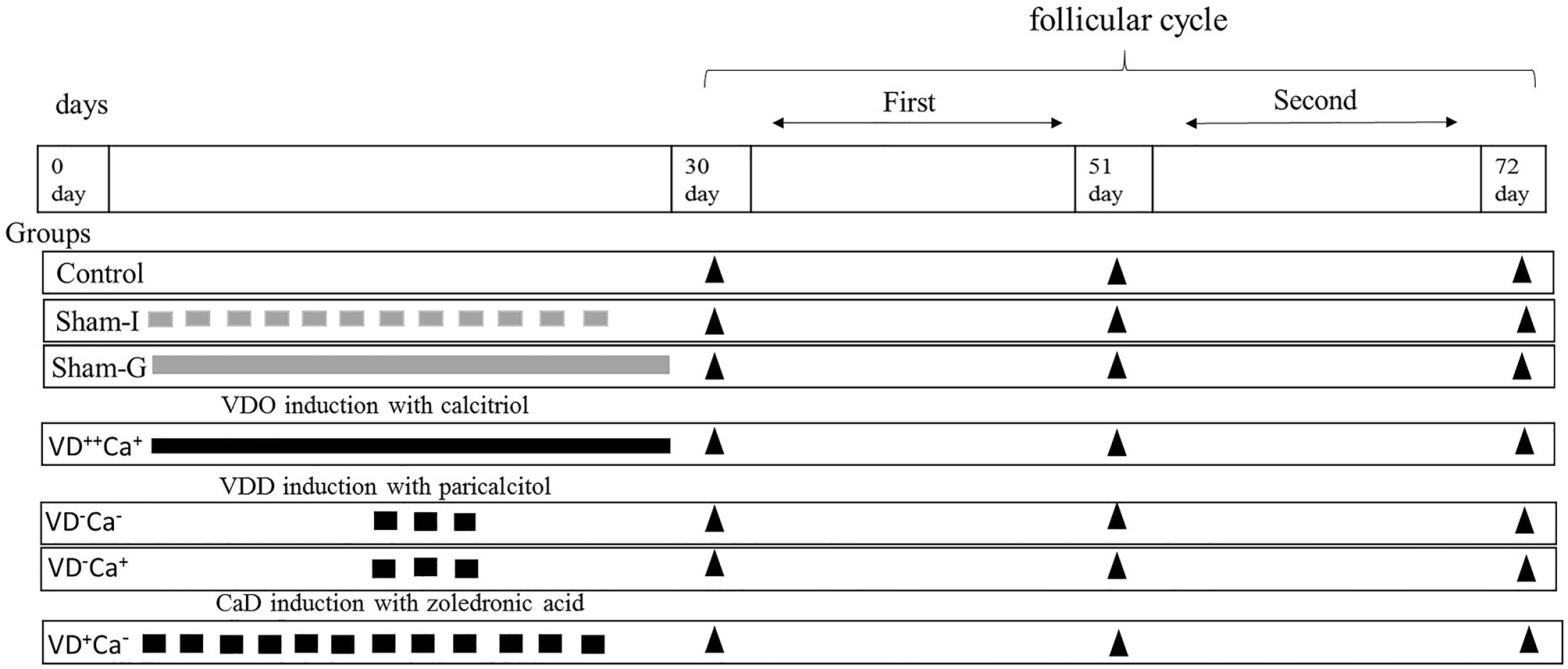
In this study, 229 NMRI female mice were used. Before randomization into groups, 5 mice were randomly selected for measurement of serum 25-hydroxyvitamin D_2_, calcium and phosphorus levels. The remaining 224 mice were then divided into 7 groups (n = 32 each). (1) control group, (2) sham groups that were injected with saline solution (Sham-I), (3) sham groups that were gavaged with saline solution (Sham-G), (4) vitamin D overdose and normo-calcemia (VD^++^Ca^+^), these mice were treated with vitamin D daily with low phosphorus and calcium diet , (5) vitamin D deficient and hypo-calcemia (VD^−^Ca^−^), these mice were treated with calcitriol and normal phosphorus and calcium diet, (6) vitamin D deficient and normo-calcemia (VD^−^Ca^+^), these mice were treated with calcitriol and high phosphorus and calcium, (7) vitamin D normal and hypo-calcemia (VD^+^Ca^−^), these mice were treated zoledronic acid with low phosphorus and calcium. The black arrowhead at 30, 51, 72 days marks the time at which 4 mice were randomly sacrificed from each group to measure serum 25-hydroxyvitamin D_2_, calcium and phosphorus levels. Grey or black rectangles marks the time and number of saline or treatment injections, respectively. Grey or black rods mark the duration that mice were gavaged with saline or vitamin D daily.

Female NMRI mice aged 4–5 weeks and weighing 25–30 g were used. They were randomly divided into control, sham and study groups. The mice were kept in cages with standard food and 12 h of light *per* day. Before allocating the mice to the different groups, 5 mice were randomly selected and their serum 25-hydoxy-viatmin D2, calcium, and phosphorus levels were assessed (Fig. 4). For the period of adaptation to the animal facility, the mice were maintained for 10 days on a standard diet. The groups of mice were as follows:

**Control group:** mice were maintained on a standard diet and were not subjected to any treatment. In this group, the diet contained 0.67% phosphorus, 1% calcium and 2200 IU vitamin D3/kg of diet**. Sham groups: Sham injection**—mice were maintained on a standard diet and injected with a saline solution, 3 times *per* week for four weeks. In this group, the diet contained 0.67% phosphorus, 1% calcium and 2200 IU vitamin D3/kg of diet. **Sham gavaged**—mice were maintained on a standard diet and were gavaged with a saline solution daily for 4 weeks. In this group, the diet contained 0.67% phosphorus, 1% calcium and 2200 IU vitamin D3/kg diet. *Please note that as the evaluation of 25-hydoxy-viatmin D2, calcium, and phosphorus levels in these two groups were identical, they were merged and were presented in the results section as one single sham group.* **Vitamin D overdose/normo-calcemia (VD**^**++**^**Ca**^**+**^**) group:** each mouse was gavaged with 1000 IU vitamin D daily for 4 weeks. In this group, the diet contained 0.4% phosphorus, 0.2% calcium and 2200 IU vitamin D3/kg of diet^35^. **Vitamin D deficient/hypo-calcemia (VD**^**−**^**Ca**^**−**^**) group:** mice were treated with 32 ng paricalcitol 3 times *per* week for one week. In this group, the diet contained 0.67% phosphorus, 1% calcium and less than 8 IU vitamin D3/kg of diet^36^. The mice were maintained under UV restriction conditions^37^. **Vitamin D deficient/normo-calcemia (VD**^**−**^**Ca**^**+**^**) group:** mice were treated with 32 ng paricalcitol 3 times *per* week for one week. In this group, the diet contained 1.25% phosphorus, 2% calcium, 20% lactose and less than 8 IU vitamin D3/kg of diet^36^. The mice were maintained under UV restriction conditions^37^. **Vitamin D Normal/hypo-calcemia (VD**^**+**^**Ca**^**−**^**):** each mice was treated with 0.02 mg of zoledronic acid 3 times *per* week for four weeks. In this group, the diet contained 0.4% phosphorus, 0.2% calcium and 2200 IU vitamin D3/kg of diet^38^.

The number of mice allocated to each group was 32. After allocation, on days 30, 51 and 72, four mice were randomly selected and sacrificed to evaluate serum 25-hydoxy-viatmin D2, calcium and phosphorus levels. The intervals between 30–51 and 51–72 days, correspond to one and two cycles of folliculogenesis, respectively. The remaining 20 mice *per* group were used for in vitro and in vivo studies. Paricalcitol (ZEMPLAR) was obtained from AbbVie (Cham, Switzerland). This compound is an analog of vitamin D2 that catabolizes endogenous vitamin D through increasing expression of CYP24A and thereby reduces the serum level of 25-hydoxy-viatmin D2. Zoledronic acid (ZOMETA) was obtained from Ronak Pharmaceutical Co (Tehran, Iran). This compound belongs to the aminobisphosphonates, one of two classes of bisphosphonates which are highly effective inhibitors of bone resorption. Vitamin D3 or calcitriol was obtained from Vitabiotics (Tehran, Iran).

Following anesthesia, cardiac puncture was performed to collect blood samples in order to evaluate serum for 25-hydoxy-viatmin D2, calcium, and phosphorus levels. Blood samples were transferred to vials (1.5-ml) and allowed to remain at room temperature for 1 h, to recover the serum fraction, the vials were centrifuged at 1800*g* for 15 min. Then the serum was frozen at − 20 °C until analysis. Serum 25(OH)D3 concentrations were analyzed using an HPLC system (Agilent Technologies, America) while calcium and phosphorus were determined using standard laboratory procedures based on radioimmunoassay (RIA) obtained from Pars Azmoon co (Tehran, Iran)^14^.

### In vivo studies

After the end of the treatment period, two female mice were mated with one male mouse (8–10 weeks old). Assessment of the vaginal plug was performed daily to ensure that mating had occurred. During the mating period and also after the mating period, mice in each group were maintained under the same conditions as described in the experimental design. The time between mating and parturition was recorded. After parturition, the number of pups in each litter was monitored. Mating ratios (i.e., the total number of females becoming pregnant divided by the total number of days the females were in the presence of a male) were evaluated as previously described by Johnson and Laura^34^. With a normal estrous cycle of 4–5 days, the mating ratio should be between 0.2 and 0.25. Fertility rate, defined as the total number of females that become pregnant and give birth to live, healthy litters divided by the total number of mating days, was similarly evaluated. In the absence of complications during pregnancy, the fertility rate should equal the mating rate. Final pup survival and litter loss were recorded at 2 weeks postpartum.

### In vitro studies

For in vitro studies, 3 replicates (3 mice each) were performed on 3 consecutive days. To induce super ovulation, 10 IU of pregnant mare serum gonadotropin (PMSG; Pregnecol, Armidale, Australia) in 0.1 ml of saline solution was injected intraperitoneally to each mouse. Two days after the first injection, 10 IU of human chorionic gonadotrophin (hCG; Pregnyl, Tehran, Iran) in saline solution was injected to induce ovulation. The mice were sacrificed 12 h later and the ovaries and fallopian tubes were dissected and transferred to HTCM (HEPES tissue culture medium) containing 10% FBS (Fetal Bovine Serum) at 37ºC. Then, the number of cumulus oocyte complexes (COCs) extracted for each ovary was recorded. A fraction of the COCs was transferred to in vitro fertilization (IVF) T6 medium where they were mixed with spermatozoa, collected from the cauda epididymides. Six hours after insemination, oocytes were cleared of cumulus cells and transferred to KSOM medium (potassium-supplemented simple optimized medium). Subsequently, cleavage and blastocyst rates were assessed at day 2 and 5, respectively. The remaining COCs were dissected and their meiotic stage was defined as follows: MII (metaphase II), GV (germline vesicle) and GVBD (germinal vesicle break down). Finally, the percentage of MII oocytes was defined for each group.

### Statistical analysis

Normality of the data was tested. Two-way analysis of variance (ANOVA) was used to compare serum 25-hydroxyvitamin D_2_, calcium, and phosphorus levels between groups and different time points. One-way analysis of variance (ANOVA) was used to compare different variables between groups. P values less than 0.05 were considered significant. For assessment of mating and fertility ratio, Dunnett’s multiple comparisons test was used.

## Data Availability

Data supporting the manuscript’s findings can be found in the manuscript.
